# AgriLogger: A New Wireless Sensor for Monitoring Agrometeorological Data in Areas Lacking Communication Networks †

**DOI:** 10.3390/s20061589

**Published:** 2020-03-12

**Authors:** Mohamed Idbella, Mariano Iadaresta, Graziano Gagliarde, Alberto Mennella, Stefano Mazzoleni, Giuliano Bonanomi

**Affiliations:** 1 Department of Agricultural Sciences, University of Naples Federico II, via Università 100, 80055 Portici (NA), Italy; stefano.mazzoleni@unina.it (S.M.); giuliano.bonanomi@unina.it (G.B.); 2 R&D Department, TopView srl, San Nicola La Strada, 81020 Caserta, Italy; mariano.iadaresta@topview.it (M.I.); graziano.gagliarde@topview.it (G.G.); alberto.mennella@topview.it (A.M.)

**Keywords:** wireless technologies, smart agriculture, precision farming, Internet of Things, agro-meteorological data, sensor, drone, microclimate monitoring

## Abstract

The use of wireless technologies in the field of agriculture, or so-called smart or precision agriculture, is considered as one of the main efforts applied nowadays to multiply the food production on earth. However, wireless sensor network (WSN) technology is still at its early development stage and its application in agriculture and food industry is still rare due to the lack of farmers’ awareness and outreach about the matter. This paper presents a new agro-sensor named AgriLogger with an aim to collect, store for long periods and transmit agrometeorological data represented by temperature and relative humidity in remote areas hard to reach and not served by telecommunication networks. The sensor exhibits long battery life, in the order of 10 years, thanks to low consumption technologies and to hardware sleep/wake up approach. It can be remotely placed on preselected sites through a customized drone. This latter, equipped with a dedicated payload, can then return on the sites where sensors have been placed, and, while hovering, wakes up the single devices and uploads their collected data through local wireless network. Field tests have demonstrated that the sensor, after being placed manually in two different positions, inside and outside a vineyard canopy, is able to collect and store successfully agrometeorological data like temperature and relative humidity. Moreover, the use of a drone potentially allows the collection of data from remote areas and, therefore, is able to provide a periodical monitoring of agro-ecological conditions.

## 1. Introduction

The industry of agriculture provides the world with biological products that are sensitive to both environmental conditions variation and applied management practices [1,2]. Therefore, it is critically necessary that farmers access the information where these variations exist in their fields, in real-time, so they can adjust their practices accordingly and promptly. The need for real-time access to climatic data helps in monitoring and facing the escalation in the frequency and intensity of potentially dangerous events such as drought, heavy rainfall, flooding and extreme temperatures. As climate change is already approved to hamper agricultural growth, it is estimated to have already reduced global yields of maize and wheat by 3.8% and 5.5% respectively [3]. Thus, the major challenge identified to be addressed is to provide farmers, through precision/smart agriculture, with the required information about the climatic conditions, in a timely fashion, instead of the traditional site-specific management practiced before. For this reason, agriculture could not be left out of the technological advances taking place worldwide in all scientific fields of research. Precision farming encompasses a large array of different technologies incorporating sensors, information systems, enhanced machinery, and decision supporting systems with a main goal of re-organizing the whole agricultural system in the direction of a low-input and high-efficiency for a sustainable agriculture [4,5]. To attain such a goal, precision farming provides the means for observing, assessing and controlling agricultural practices from the quotidian herd management through horticulture to field crop production. The industry of agriculture is, at present, capable of collecting more inclusive data on different production controlling parameters in both space and time [6]. It provides a decision support system for delivering a large vision about the possible treatments, whether field-wide or for only specific parts of the field, and the means for taking the proper reaction according to the data collected [7]. Nowadays, the quick evolvement of technology in the fields of IoT (Internet of Things), UAS (Unmanned aerial systems), low power devices and sensors, is opening up new frontiers in the agricultural applications. In this regard, it is possible to convert the traditional farm approach into the “Smart Farm” philosophy, which allows the access for more accurate monitoring of crop development and health status with adequate temporal, spatial, and spectral resolutions [8]. Furthermore, a UAS platform with proper sensors, based on wireless sensor network (WSN), is becoming a common combination in this field of research offering a flexible, convenient, and cost-effective way to provide enquiries about the desired observations on agricultural parameters [9]. In this regard, Di Francesco et al. [10] have provided a full literature survey about the use of WSN along with mobile vehicles generally, including robots, terrestrial vehicles, and also UAS. Moreover, Zhan et al. [11] have moved a step further and proposed an optimization solution to jointly ameliorate the WSN wake-up schedule with the drone’s pathway in order to minimize the energy consumption.

UAS are aerial vehicles that can be presented differently depending on their shapes and sizes, and can be remotely controlled or can fly autonomously throughout a software-controlled flight on the basis of a GPS system [12]. The use of a light composite materials in the made-up of UAS helps to reduce their weight and increase their position-changing capability with a strong potential to fly in high altitudes depending on data collection needs. Due to the various navigation systems and recording devices that can be possibly embedded in the UAS, they can travel inaccessible areas providing the real-time monitoring, through dedicated payloads, of the state of health of the crops and therefore quick raw data of different agro-metrological parameters [13,14,15]. Sensor networks, in conjunction with UASs, are often implemented in research settings to expedite the process of data collection and to increase the breadth of data that may be collected over a geographical area. This conjunction is applied normally to monitor marine-coastal environments for environmental metrics data collection, such as water temperature, salinity and pH. Moreover, it is potentially useful to monitor agrometeorological data in remote areas (i.e., vineyard and olive cultivation in steep slopes, forests on cliff, large trees in urban environment, etc.), and also to monitor greenhouse atmospheric gas concentrations and many other examined applications in mapping, feature detection, and monitoring wildlife.

IoT is defined as the worldwide network of interconnected objects (devices, mechanical and digital machines, animals or people) uniquely addressable based on standard communication protocols, that are provided with the ability to transfer data over a network without requiring human-to-human or human-to-computer interaction [16,17]. In the agricultural field, IoT allows us to increase the monitoring points of agro-meteorological parameters and to remotely control the agro-actuators, with reduced costs compared to the traditional approach. It owes much of its success to the widespread distribution of Internet communication networks (without this, it would not be applicable), even in agricultural areas.

In Italy, there are still large portions of territory, mostly in the Apennines mountain chain, not served by communication networks due to low population density. These areas host agricultural and forestry activities located at different altitudes, which is difficult to monitor. The use of WSN for monitoring climatic changes such as temperature degree and relative humidity in agricultural fields has been studied considerably but substantial applications are still rare [18,19,20]. Therefore, new solutions are needed in order to monitor constantly and remotely the data of interest, overcoming the barriers given by lacking communication networks and difficulties to reach hostile territories. For this reason, this work focuses on the properties of a new sensor called “AgriLogger”, coupled with an UAS system, capable to collect, store for long periods and transmit agro-meteorological data, presented by temperature and relative humidity, in remote areas that are hard to reach and not served by telecommunication networks.

## 2. Material and Methods

### 2.1. AgriLogger Structure

**Figure 1:**
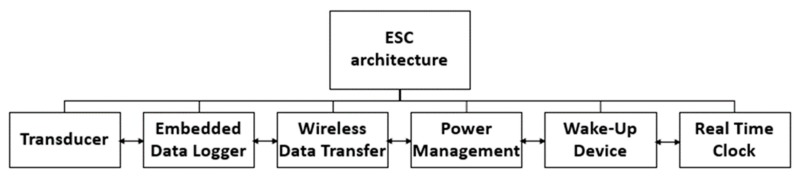
ESC™ (Easy Sense & Connect) architecture used for AgriLogger manufacturing.

The system belongs to a family of battery-powered sensors able to collect, hold and transfer, through standard IoT policy, the measurements of two essential environmental parameters: temperature degree and relative humidity. The agrosensor has been derived from ESC™ (Easy Sense & Connect) architecture (Figure 1), which is able to reduce the energy consumption through the suppression of the IoT network interface as LoRa (Long Range) or NB-IoT (Narrow Band IoT). It is formed of a transducer, a data logger based on low power embedded microcomputer family designed by Nordic NRF52832 and supported by Cypress FM24V05, containing 512 Kbit of non-volatile memory and employing an advanced ferroelectric process, which guarantees data retention for several years. Moreover, a wireless data transfer represented by BLE (Bluetooth Low Energy) transceiver with PCB printed antenna which transfers the collected data to the requesting host. a real-time clock based upon MCP79412 Microchip, and supported by a 32 kHz crystal in order to take trace of elapsed time in terms of fiscal year, month, day, hour, minute and second. The clock is set through a smartphone APP, supplied with the sensor, at the time of battery installation. Subsequently, a wake-up device is modulated according to the forecasted application. Each device is limited to waking up at a certain interval of time, taking the data and storing it in an internal memory. The awakening of the device is driven by the internal real time clock, and the process can be launched whether by using a reed relay throughout approaching a magnet to one of the ends of the external box, which reactivates the internal microcontroller, allowing Bluetooth LE connectivity. Alternatively, a directive radio wave transmitter enabled on the host, in our case a drone, can wakes up the sensor. The maximum distance between the radio wave transmitter and the awaking circuit is about 20 m. The waking-up circuit embedded in the sensor harvests energy from the radio wave. AgriLogger can connect to gateways enabled for the connection. If there is a wakeup call but there is no gateway nearby the device returns to a deep sleep state. Furthermore, the device structure contains a power manager which regulates the embedded battery pack (2xAA alkaline batteries with limited self-discharge) and provides sleep/awake status for the device.

A series of capacitors are charged to reach a certain level of electrical voltage used as an input for the microcontroller that wakes up and enables Bluetooth LE connectivity.

### 2.2. AgriLogger Composition

**Figure 2:**
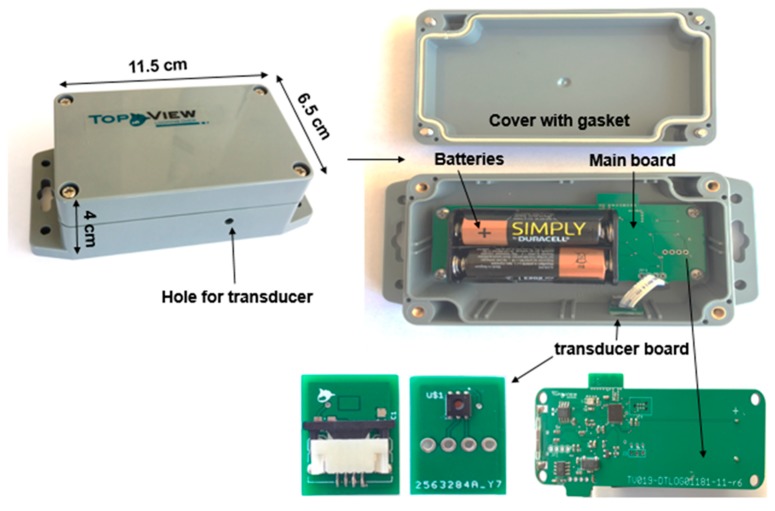
Schematic illustration of the data logger high temperature box (11.5 cm length × 6.5 cm width × 4 cm height × 190 g weight), open and closed with the identification of the inner sealed transducer and its outside hole on the box, along with the main board and the batteries as energy source for active data collection.

The sensor has been fabricated by merging two printed circuit boards, the main system board and the transducer board which changes upon application (Figure 2). The main system broad hosts the two AA batteries on bottom side and its design is based on Nordic technology NRF52832, which embeds a BLE interface too. The transducer broad aims to measure air temperature and relative humidity and it is built around Silicon Labs device SI7006-A20. Both boards have been placed inside a sealed box (IP67) in order to resist weathering, ensuring the direct contact between the temperature/humidity sensor with the ambient air, while protecting it from rain. For this purpose, a small hole (20 mm) has been provided on the box, where, in its inner side, the sensor is located and sealed to the wall, while the external side of the hole cylinder has been covered with a piece of Gore-Tex, a water repellent fabric. The prototype box for the high temperature-Sensor version is shown in Figure 2.

### 2.3. Unmanned Aerial System: Drone

**Figure 3:**
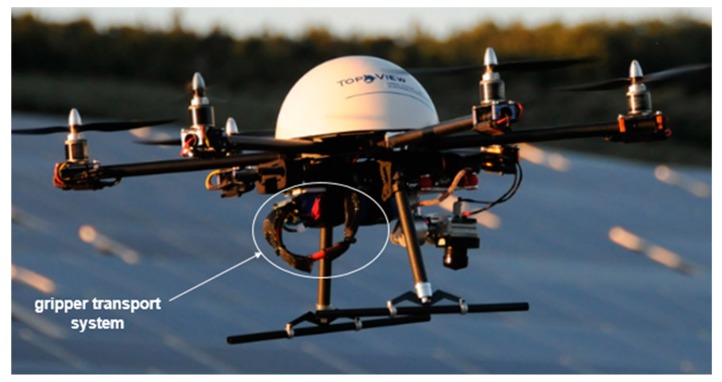
Illustration of the gripper transport system of the drone served to pick and release the sensor on the field.

**Figure 4:**
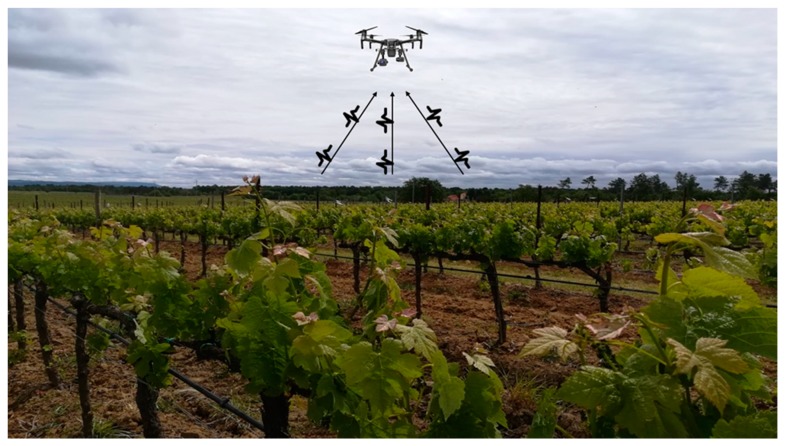
Data collection of HT box sensors from a vineyard using a far wake-up device represented by a drone.

The drone is equipped with a payload able to transport and deliver the sensor upon the preselected location, with a smooth release on the ground via the gripper transport system (Figure 3), that serves as well for recuperating the sensor after the end of the recording time or to change the batteries. It is composed by a standard unmanned aerial vehicle compliant along with the UTM (Unmanned Traffic Management) services allowing to fly in BVLOS mode (Beyond Visual Line of Sight). The payload embeds two main features, RF transmitter with directive antenna in charge of the resonant circuit stimulation (wakeup device) inside the sensor lying on the ground, and Bluetooth Low Energy (BLE) transceiver enabling the connection inside the sensor after the wake-up step. Thus, the host in the payload acts as a client and requests the upload of stored data (Figure 4).

Two versions of drone payload can be provided depending on the “Pick & Release” feature and the integration with the drone tightly or loosely coupled. The full featured version fitting on middle size drones, and the light version that can be installed on small drones.

**Figure 5:**
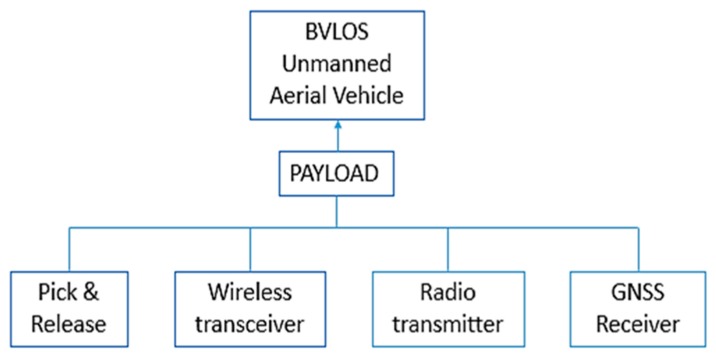
Full featured drone payload block diagram representing the main devices inside.

The full featured payload needs to be connected with the drone, by both electrical/mechanical and logical connection (proprietary DJI SDK or open Pixhawk platform). Inside the full featured payload, the main devices are represented by a gripper to transport and release the sensor in the preselected location, a host computer on board able to upload, through BLE wireless local network, data collected and stored in the sensor. Moreover, a radio transmitter, equipped with a directive antenna pointing down, to be enabled on the site where sensor is supposed to lie; and a GNSS (Global Navigation Satellite System) receiver, aimed to identify the geographic position to place the sensor and to retrieve data when needed (Figure 5).

In easy-to-reach field crops or orchards, the sensors could be placed manually on the site by an operator so that the payload can be reduced in dimensions and weight (no pick and release function) and can be installed on small drones. In this case, the payload is a small board, with its own embedded battery, including both the gateway (BLE to memory card) and the wake-up device. No electrical or logical connection are provided between the payload and the drone, only a simple mechanical connection is provided by Velcro or clamps.

### 2.4. Gateway BLE

**Figure 6:**
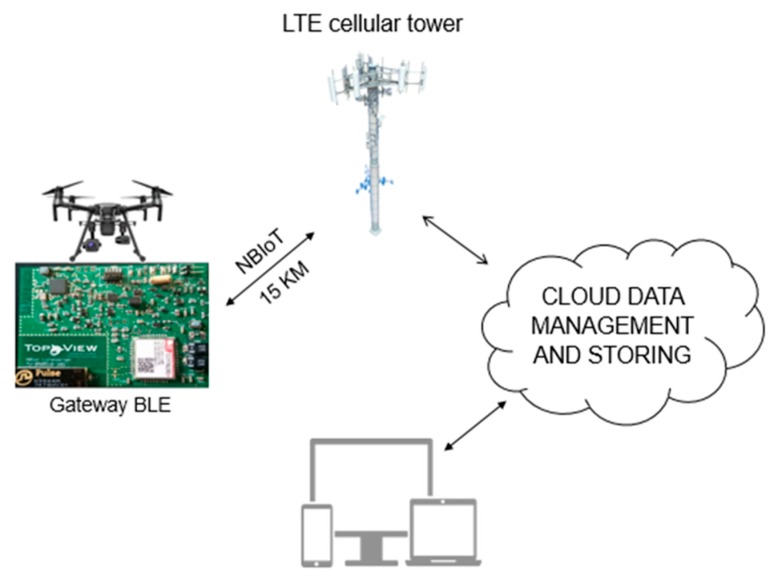
Schematic illustration of Data Flow from the AgriLogger gateway to the data receiver (computer, smartphone) throughout LTE cellular tower.

BLE Gateway is defined as the device that acts as a bridge between the local network and the internet, and is placed on board of the drone. It wakes up the sensor installed on the ground by enabling the Bluetooth LE. The awakening is due to a radio frequency signal that starts from the gateway and ends at the sensor, which enables Bluetooth LE for a successful connection. The gateway requests reception of the stored data in the AgriLogger memory. The latter starts sending data and returns to a state of deep sleep after the end of data transmission, and continues with log activity. The gateway takes the received data and starts the transmission of small packets to the cloud management service. The data transmission by the gateway requires the NB-IoT network. NB-IoT is an innovative network dedicated entirely to the IoT, in particular, for low data traffic systems. This network is public and uses LTE cells, the same ones that were used for GSM mobile phones years ago. All data passes through a network operator which in turn directs them to a final IP. The coverage of this network is almost 100% of the national territory. The main feature is that the Gateway can also be 15 km from the nearest LTE cell (Figure 6).

### 2.5. Field Test of AgriLogger on a Vineyard

**Figure 7:**
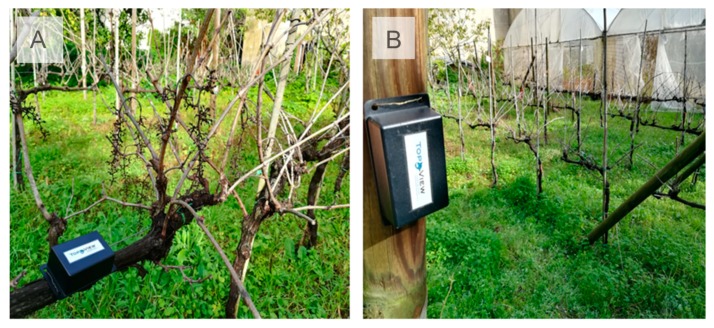
Pictures of AgriLogger in the vineyard: (**A**) inside canopy, (**B**) outside canopy. The pictures were taken in the winter to clarify the position of the sensor inside the canopy without leaf cover.

In order to evaluate the reliability of the system and the quality of data collection under field conditions, two sensor devices were placed manually inside a vineyard at the botanical garden of the agricultural faculty of Portici (Federico II university of Naples). Each sensor collects and stores on its own data-logger, one sample per hour of air temperature and relative humidity. Data collection was made during two different seasons, during the summer 2019 from 12 to 27 August, and during the fall from 1 to 19 October. The purpose was to characterize the microclimates outside and inside the canopy of an agricultural vineyard during these two different seasons (Figure 7). Monitoring microclimatic condition in vineyards is pivotal for an efficient plant protection of diseases such as powdery mildew (caused by *Erysiphe necator*) and downy mildew (caused by *Plasmopara viticola*) [21]. The sensors were placed manually due to the easy accessibility of the study site; however, the data were collected using a drone as described previously. The use of a drone to collect data is to demonstrate the reliability of the WSN-UAS combination in the agricultural field, and to show the applicability of these systems to collect data in areas that are hard to reach.

## 3. Results and Discussion

### 3.1. Battery Endurance

The main characteristic of ESC™ architecture is the extended working life without changing batteries. This main power derives both from the speeded-up tests in the lab and from the calculations that are summarized below. For AgriLogger, AA Duracell Plus battery was found to be the best option in the market due to its strong characteristics, with a high voltage of 1.5 V, a high capacity of 2000 mAh and a very low self-discharge of 0.5% per month (Table 1).

AgriLogger sensor was made in a way that each device inside the whole structure requires a negligible amount of power for each operating mode, the highest consumption is conducted by the data logger block that needs 0.1 μA for the sleep mode, 400 μA for uploading and 400 μA for sampling. The minimum consumption among the inner devices is drained by the wake-up circuit that demands 0.8 μA for each operating mode (Table 2). Further, ESC power consumptions are declared in Table 2, where the energy request of each block of the architecture is listed for the provided operating modes.

Data reported in Table 1 and Table 2 allow the calculation of device life, without changing batteries, in the conditions shown in Table 3. The power consumption is given knowing that data upload is done once each week. The overall consumption of current during the whole week is calculated by considering a constant sink of sleep current, plus the amount requested during the sampling phase, plus the current needed during the upload of collected data made once in a week. By a simple calculation we can estimate that the life of the device is longer than 10 years (532 mAh requested for that, Table 3). Therefore, the true limit is the quality of battery. Duracell, as a standard AA battery for instance, assures a limited self-discharge (0.5% per month) which should assure a battery life of more than 16 years. However, the expiration date marked on the products is always less than 10 years. In any case we consider that a duration of 5 years for this ESC™ device is enough for all the foreseeable applications.

### 3.2. Field Test

**Figure 8:**
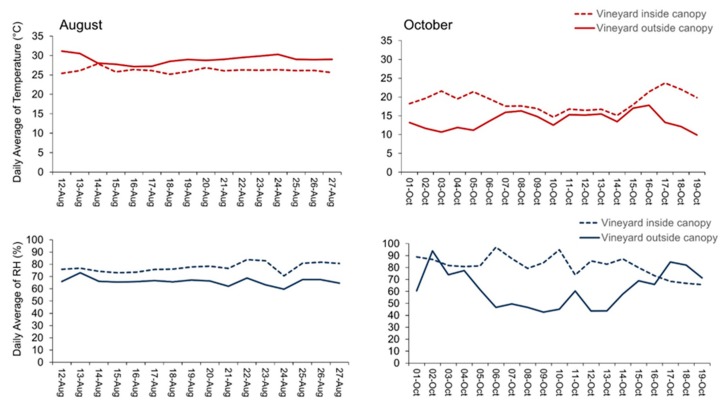
Daily average of temperature (°C) and relative humidity (%) recorded by agriLogger sensors positioned inside and outside a vineyard canopy in August and October.

Our results have demonstrated that the magnitude of the meteorological parameters was less variable in the hotter month than in winter. During August, the daily average temperature remained relatively stable, around 30 °C outside the vineyard canopy and 25 °C inside, during the whole recording (Figure 8). During October, the temperature daily average decreased to low values for both positions, with a daily minimum of 10 °C and 18 °C, and a daily maximum of 17 °C and 22 °C, outside and inside the canopy correspondently. Accordingly, the daily average of relative humidity (RH) during August recorded values around 70% outside the canopy and around 80% inside, with a daily maximum of 73% outside the canopy and a daily maximum of 85% inside. However, during October, RH daily average outside the canopy registered very low values arriving to a minimum of 40% while inside the canopy recorded high values arriving at a maximum of 98%.

**Figure 9:**
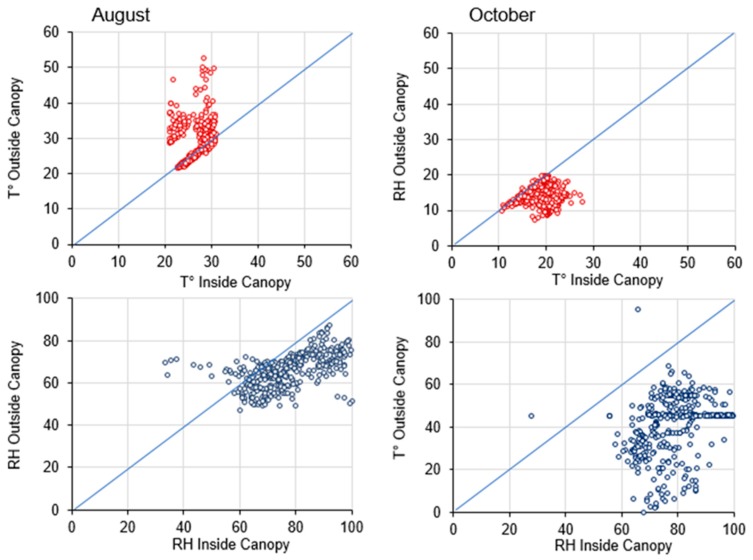
Scatter plot of temperature (°C) and relative humidity (%) inside and outside a vineyard canopy in August and October. Data refer to values recorded every hour during the whole recording month. Values over the 1:1 indicate that the variable has the same values in the different conditions, while values above or below the line highlight that the temperature or relative humidity are higher or lower outside the canopy, respectively.

The sensor has recorded high values of temperature outside the canopy, during August, compared with the inside. During October, the temperature values recorded were higher inside the canopy rather than outside (Figure 9). Regarding the humidity percentage, inside the canopy registered more humid values compared to outside during both months (Figure 9).

The impact of a plantation canopy on understory microclimate is directly and indirectly associated with the presence of the canopy structure presented by stems, branches, and leaves. This structure reflects and absorbs a significant part of the solar radiation during the day, which results in allowing less energy to reach below the canopy [22]. For this reason, the temperature inside the canopy was less than outside in our assay. However, the degree of absorption is broadly determined by stand structure and species composition [22,23,24,25]. Moreover, the differences of average RH recorded during October by the sensors can be explained by the fact that the canopy provides resistance from the mixing of air and the exchange of air with the surrounding atmosphere [22,26]. In addition to the effect of evaporation from soil and wet plant surfaces along with the transpiration displayed by leaves, which all contribute to adding water to the air while cooling and therefore lowering the water holding capacity of the air, which causes thus the general increase of relative humidity below canopy [22,27].

In line with our results, many previous studies have confirmed the behavior of microclimatic conditions during different seasons inside and outside a plantation canopy. For instance, Miranda and his colleagues [28] have showed that the temperature outside cacao canopy always remained higher than inside during summer. During winter, temperatures outside and inside were not observed to change very much. On the other hand, relative humidity inside the cacao canopy was moderately higher during the summer and extremely higher during winter compared to the outside. Moreover, a recent study on a pine forest demonstrated that the plantation canopy lowered daily maximum air temperature by up to 5.1 °C and increased daily minimum relative humidity by up to 12.4%, which are ranges that will significantly affect growth of understory tree seedlings. However, the moderating capacity was generally stronger in summer than in winter [29].

Our results prove that AgriLogger, in the field, was able to collect and store, for long periods estimated for more than 10 years, and consistently, agrometeorological parameters data in a successful way that would be of great profit for agriculture. Such data provides real-time information about the weather, which is one of the key components that controls agricultural production, because it affects crops at all stages of their growth cycle. In some cases, more than 80% of the agricultural production variability is due to the changeability and undredictibility of weather conditions [30,31,32]. In addition, the success of mostly every operation within each farming system depends on the immediate former weather, the prevailing weather as well as the weather of the next few hours or days [33,34,35,36]. Nowadays, modern farming has already acknowledged that timely availability and appropriate use of agrometeorological information are vital to successful farming operations. Air temperature, as an example, is considered as the main weather variable that regulates the rate of vegetative and reproductive development [37]. Therefore, getting access to such a variable, helps in applying the required treatments in real-time and thus in profitably regulating farm production, and in disaster risk reduction processes as well [38].

## 4. Conclusions

Wireless sensor networks, based on the Internet of Things, for monitoring agro-climatic parameters in real-time, have been developed to benefit farmers, agronomists, and meteorologists. Different blocks were combined together to perform efficient roles of interconnectivity, interoperability and remote monitoring on a real-time basis. AgriLogger is designed and implemented to realize modern precision agriculture, it was tested and deployed into the field for sensing climatic changes throughout different seasons of the year, which therefore will allow farmers to reliably collect data from locations previously inaccessible due to lacking communication networks. Such a system can be easily installed and maintained. This paper successfully applies the wireless sensor networks on agro-ecology fields by investigating environmental situations. However, the big challenge facing AgriLogger functioning relies on the fact that, although the battery consumption could keep the sensor working for more than 16 years, the collecting phase lasts only 2 years due to the limit imposed by the size of the internal memory of the device. Moreover, some ecological effect can cause data transmission failure in WSN since it covers only short ranges. Therefore, more investigations and adjustments are needed in order to finalize the industrial use of AgriLogger.

## Figures and Tables

**Table 1:** Standard Battery characteristics with the appropriate condition associated with each parameter value.

AA Duracell Plus Battery Specification		
Parameter	Value	Conditions
Voltage	1.5 V	1A load
Capacity	2.000 mAh	10 m A load
Self-Discharge	0.5% Per month	20 °C

**Table 2:** ESC different structures with the power consumption of each device according to the operating mode in action.

Device Elements	Operating Mode		
	Sleep	Upload	Sampling
Microcomputer	2.3 μA	7.8 mA	2.0 mA
Data logger	0.1 μA	400 μA	400 μA
Real-time clock	1.2 μA	1.2 μA	400 μA
Wake-up circuit	0.8 μA	0.8 μA	0.8 μA
Power module	0.0 μA	100 μA	0.0 μA
Transducer module	60 nA	150 μA	150 μA
Cumulative current	4.5 μA	9.65 mA	2.95 mA

**Table 3:** An example of working conditions of AgriLogger sensor depending on the preferred settings to be applied.

Setup Values		
Parameter	Value	Notes
Sampling time interval	1 h	-
Sample collection time	2 s	Average value of 10 samples
Upload time interval	168 h	1 week
Upload duration	10 s	9600 baud
Overall consumption	1.04 mAh	In one week
